# Supplementary material for: Conformational changes in intact dengue virus reveal serotype-specific expansion
Source: Nat Commun. 2017 Feb 10;8:14339. doi: 10.1038/ncomms14339 (PMC5309819; doi:10.1038/ncomms14339)
Supplement: Supplementary Information — Supplementary Figures, Supplementary Tables, Supplementary Notes and Supplementary References [file ncomms14339-s1.pdf]

Supplementary Figures:

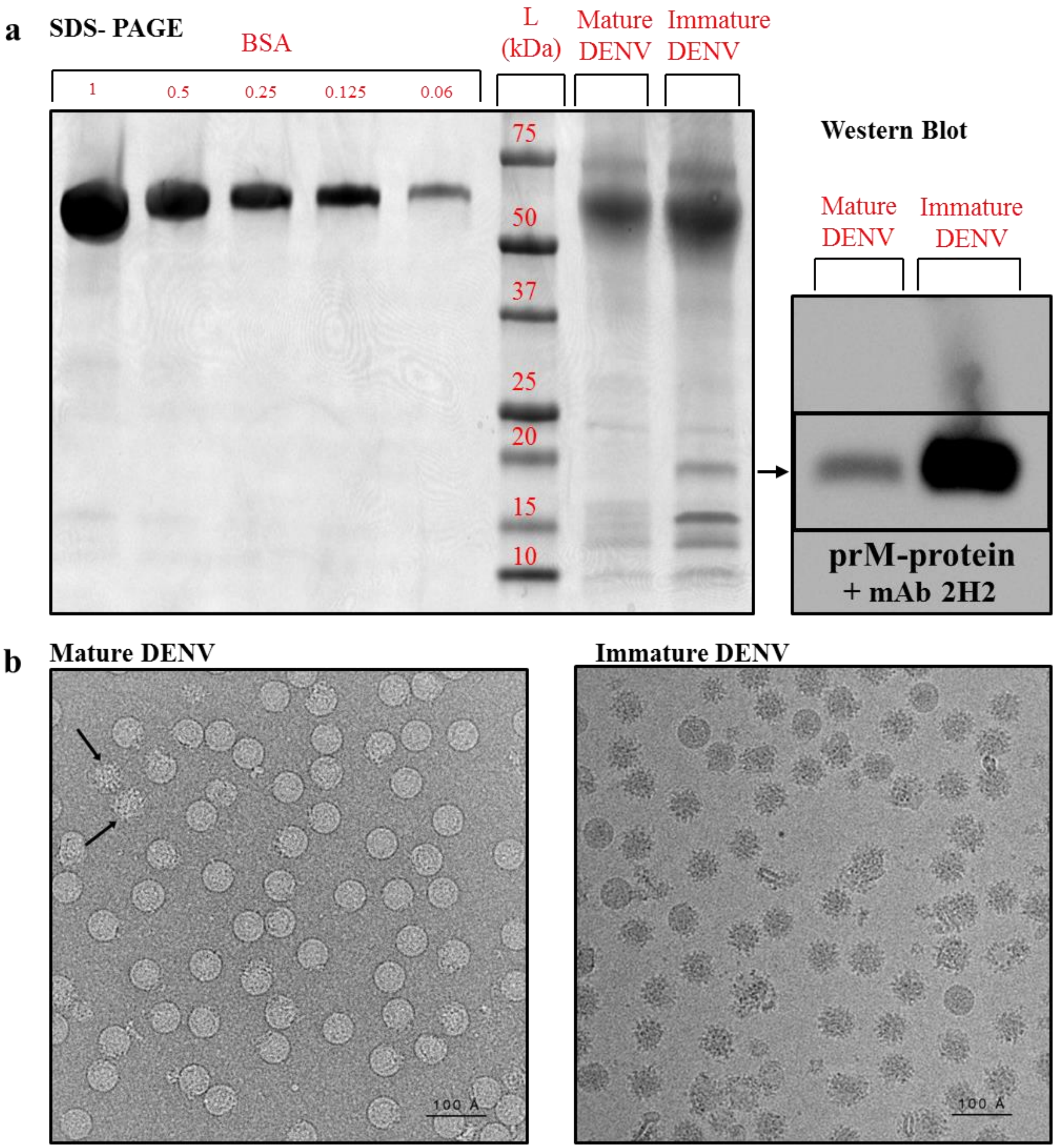

c

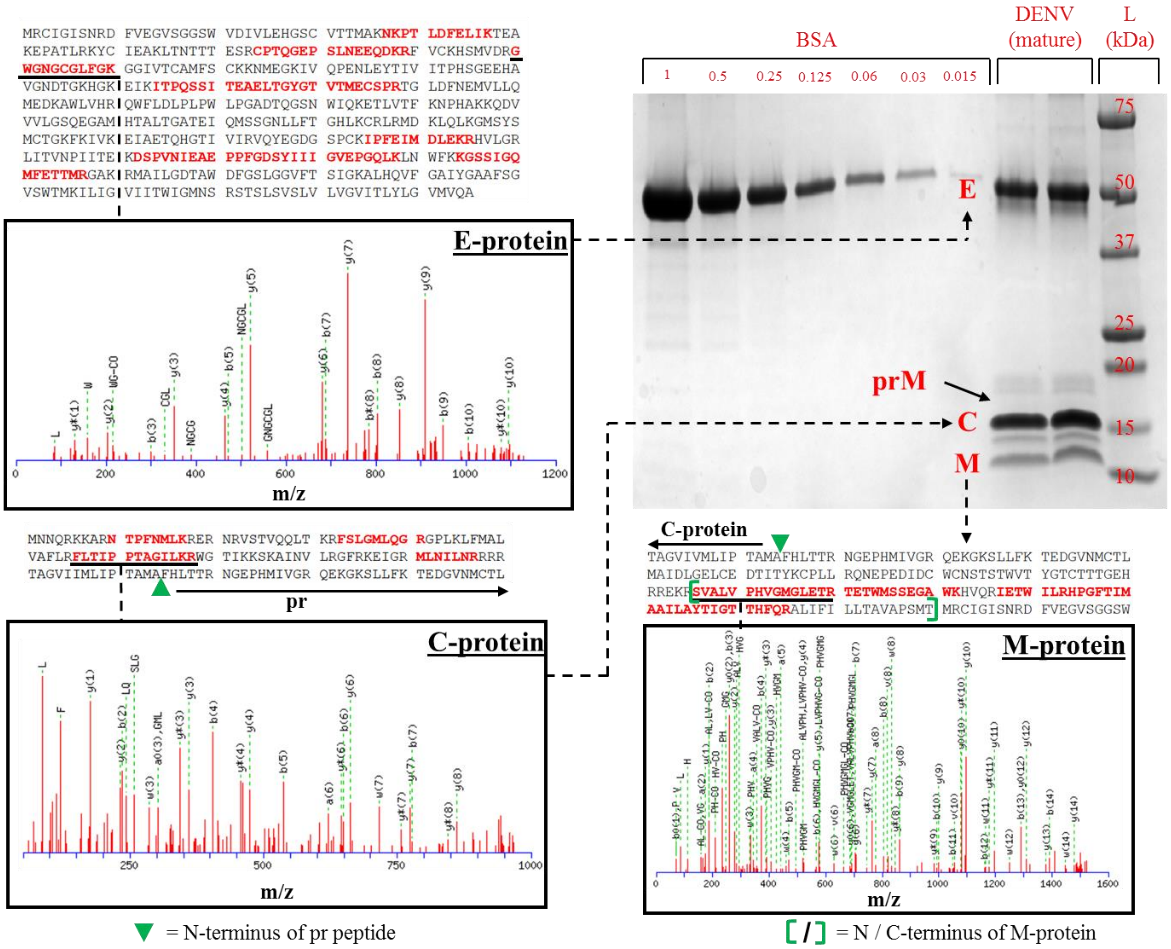

Supplementary Figure 1: **a)** SDS PAGE of mature and immature DENV (lanes 7 and 8 respectively) and BSA standard 1, 0.5, 0.25, 0.125 and 0.0625 mg/mL in lanes 1 – 5. Size of BSA and DENV structural proteins were estimated from a reference protein ladder (L) (lane 6). **i**Western blot identification of prM-protein in mature and immature DENV samples with anti-prM-protein mAb (2H2). A faint prM-protein band in the mature DENV preparations indicate very low amounts of immature DENV in the mature DENV samples. **b)** Cryo-EM micrograph of purified DENV samples at 28 °C with scale bar representing 100Å. Only 2 immature DENV (indicated by black arrows) were observed amidst the vast majority of mature DENV particles. Cryo-EM micrograph of purified immature DENV is included for reference with scale bar representing 100Å. **c)** SDS-PAGE gel of purified mature DENV (lanes 8 and 9) and BSA (lanes

1-7). Quantity of viral particles were estimated by comparing the band intensity of E-protein with the band intensity of BSA standards ranging from 1 – 0.015 mg/mL. The purified DENV samples contained mainly mature particles as only very faint prM-protein band was observed. Distinct bands observed from SDS PAGE of mature DENV were identified to be only E-, C-, and M-proteins by mass spectrometry. Trypsin proteolysis peptides identified by MS analysis and sequenced by MS/MS fragmentation corresponding to each proteins are shown in red. MS/MS fragmentation ions of each representative peptide (underlined) for each proteins are shown. No tryptic peptides corresponding to pr peptide was detected in any of the three distinct bands.

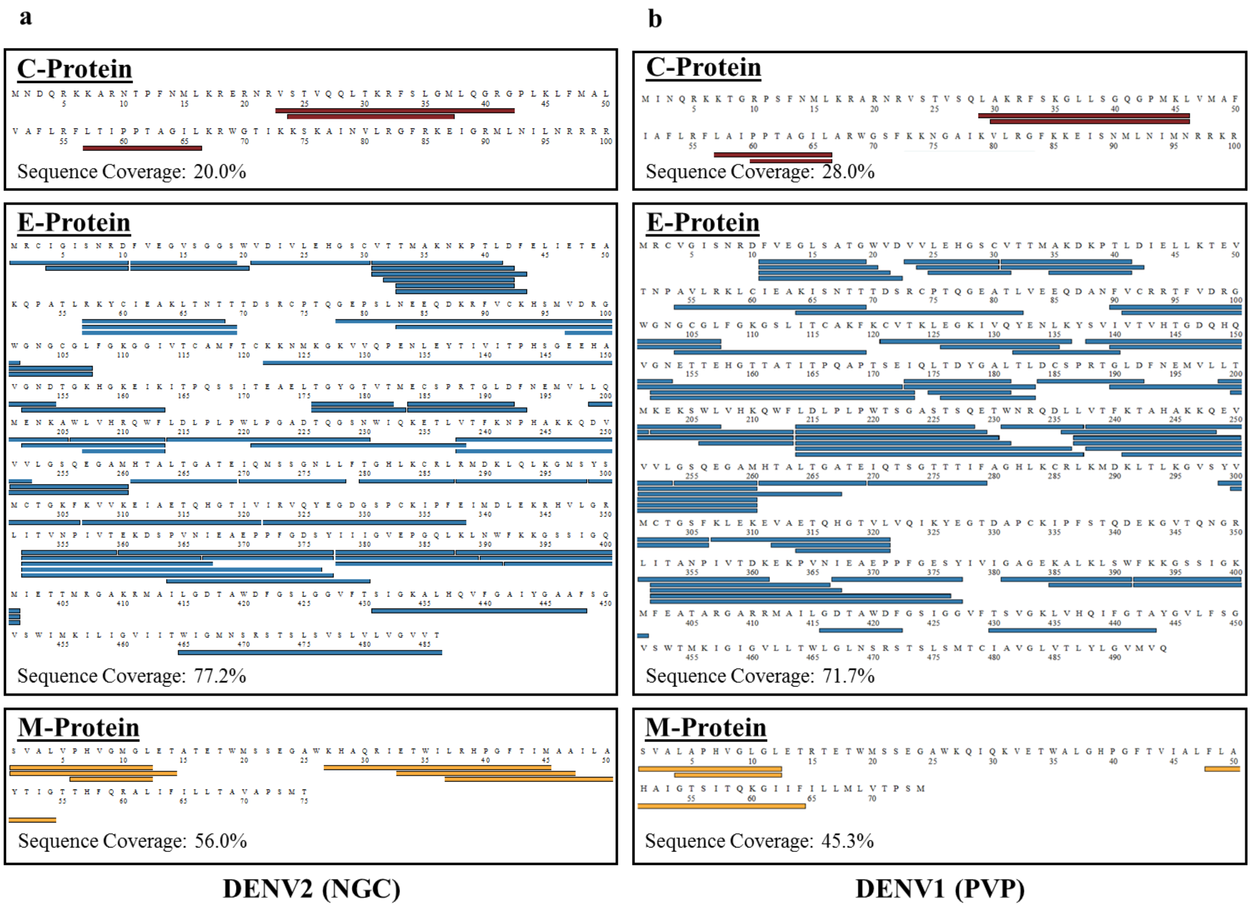

Supplementary Figure 2: **Primary sequence coverage map of pepsin proteolyzed peptides of DENV2 and DENV1 proteins.** Pepsin fragmentation peptide map for C, E- and M-proteins of DENV2 **(a)** and DENV1 **(b)**, with peptides from C-, E- and M-proteins represented by red, blue and yellows bars, respectively.

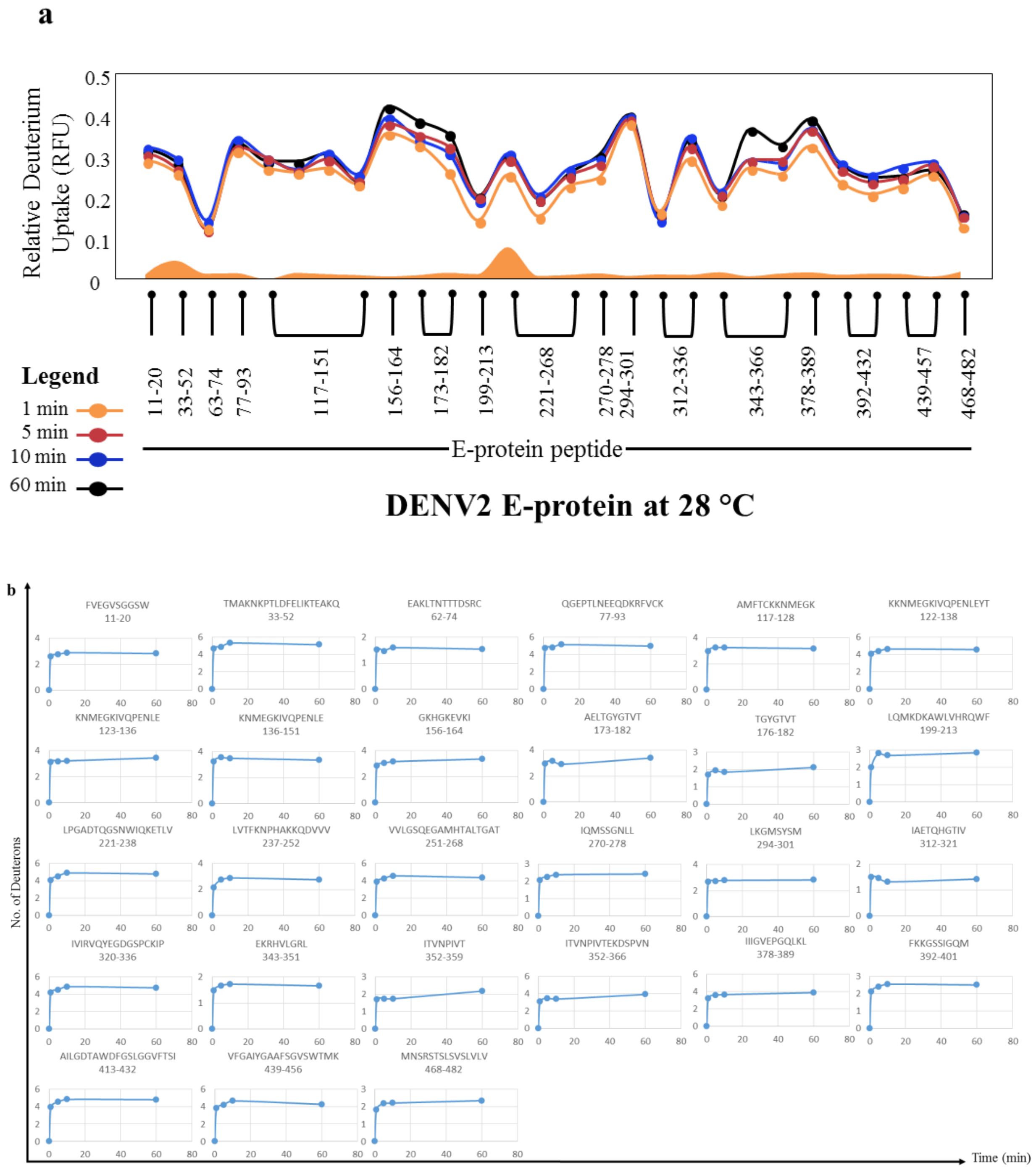

Supplementary Figure 3: **Deuterium exchange and uptake plots for pepsin-proteolyzed peptides of DENV2 E-protein. a)** Relative fractional deuterium uptake (RFU) across viral E-protein at 28 °C for time points (1, 5, 10 and 60 min). Relative fractional deuterium uptake (RFU) (Y-axis) for all peptides (X-axis) across time-points 1, 5, 10 and 60 min are shown.

Standard errors for each peptide are shown as shaded regions along the X-axis and are color coded the same as the RFU plots. The standard error for a given peptide represents the sum of the single sigma standard deviations over all the time-points from all the (minimum three) independent HDXMS measurements. These show negligible time-dependent increases in deuterium uptake from 1 to 60 min of deuterium labelling, at 28 °C. **b)** Deuterium uptake kinetic plots for all peptides after 1, 5, 10 and 60 min of exchange at 28 °C. Most peptides show a constant deuterium exchange from1 min up to 60 min. Amino acid sequences and corresponding residue numbers for all peptide are indicated for each plot.

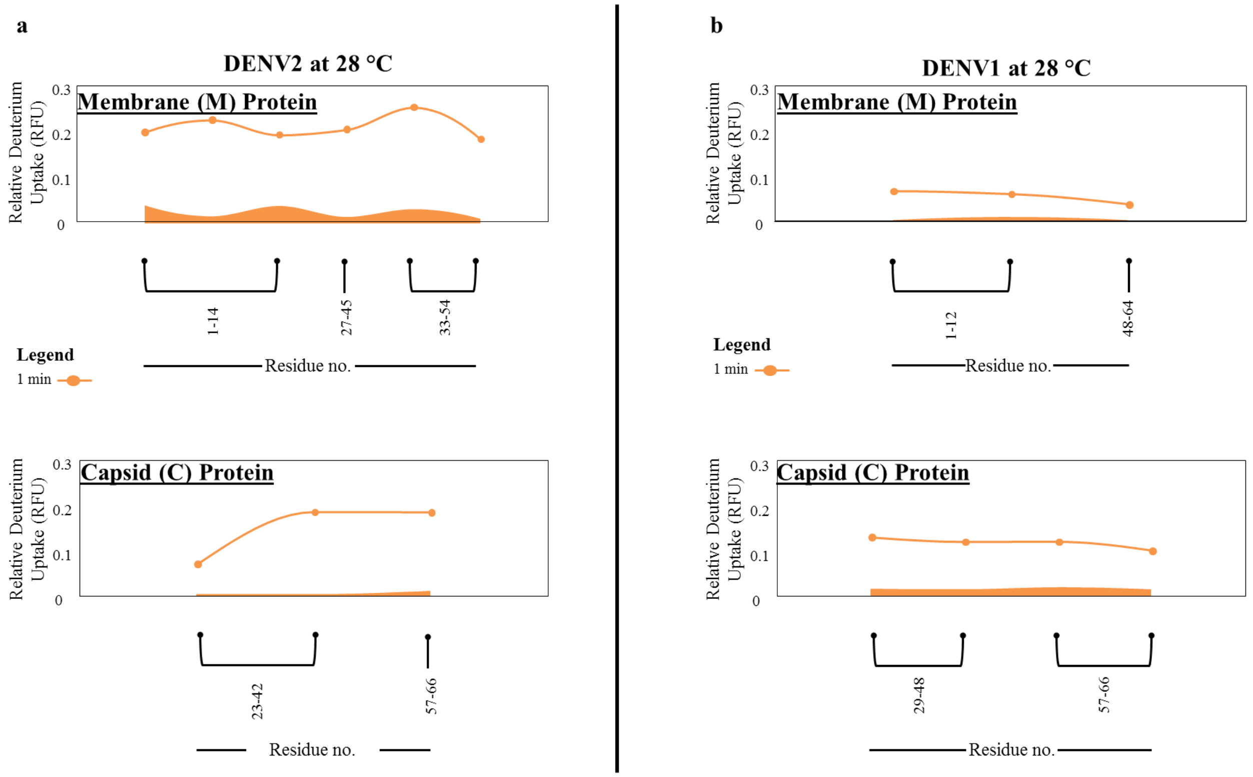

Supplementary Figure 4: **Comparison of relative fractional deuterium uptake of M- and C-protein from DENV2 and DENV1 after 1 min of deuterium exchange at 28 °C.** Relative fractional deuterium uptake (RFU) (Y-axis) for all peptides (X-axis) of M- and C-protein from DENV2 **(a)** and DENV1 **(b)**, at 1 min of deuterium exchange, is represented on a modified mirror plot. M- and C-protein from DENV1 display lower relative deuterium uptake compared to DENV2 at 28 °C. Standard error for each peptide is shown as shaded regions along the X-axis and color coded the same as the RFU plots. The standard error for a given peptide represents a single sigma standard deviation of three independent HDXMS measurements.

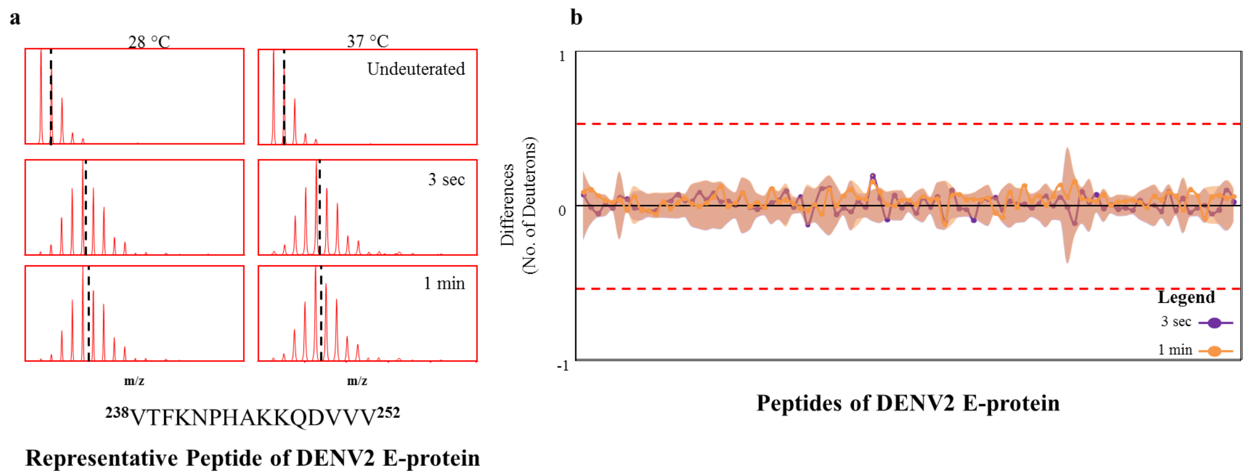

Supplementary Figure 5: A 2.3X fold estimated increase in estimated rates of intrinsic amide H/D exchange rates for peptides is undetectable between 28 and 37 °C. a) Deuterium uptake of a representative peptide generated by pepsin proteolysis of unassembled E-protein prior to deuterium exchange is shown. E-protein pepsin proteolysed peptides were subjected to deuterium exchange for 0 s (undeuterated), 3 s and 1 min at 28 and 37 °C. Black dashed lines indicate position of the centroids of the mass spectral envelopes in each condition. **b)** Deuterium exchange difference (Y-axis) of all E-protein fragment peptides, listed from N- to C- terminus (X-axis), after 3 s and 1 min of deuterium exchange between 28 and 37 °C. Differences in deuterium exchange above 0.5 D are considered significant (red dashed line). The individual standard error of each conditions is calculated as standard deviations observed across all the HDXMS measurements from three independent HDXMS experiments. The standard error for a given peptide in the difference plot represents the sum of such single sigma standard deviations, in three independent HDXMS measurement, of each of the two conditions being compared and colored same as the difference plot.

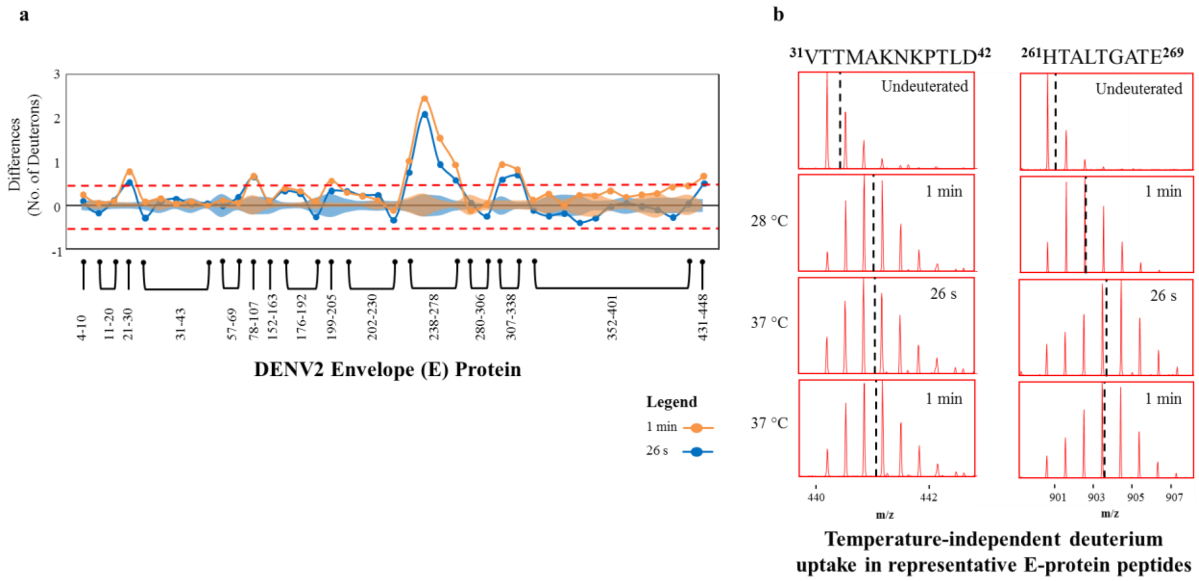

Supplementary Figure 6: Loci displaying temperature-dependent increases in deuterium uptake are similar for deuterium exchange of intact DENV2 particles at 26 s at 37 °C and for 1 min at 28 °C. a) Differences in number of deuterons between deuterium exchange of DENV2 at 37 °C for 26 s and 1 min of deuterium exchange at 28 °C. Differences in deuterium exchange above 0.5 D are considered significant (red dashed line). Standard error for each peptide is shown as overlapping shaded regions along the X-axis according to the difference plot. The individual standard error of each condition is shown as standard deviations observed across all the HDXMS measurements from three independent HDXMS experiments. The standard error for a given peptide in the difference plot represents the sum of such single sigma standard deviations of each of the two conditions being compared. **b)** Representative mass spectra of peptides displaying temperature-dependent and temperature independent deuterium exchange after 26s or 1 min of deuterium exchange at 28 or 37 °C. Black dash lines represent position of the centroids within the mass spectral envelopes.

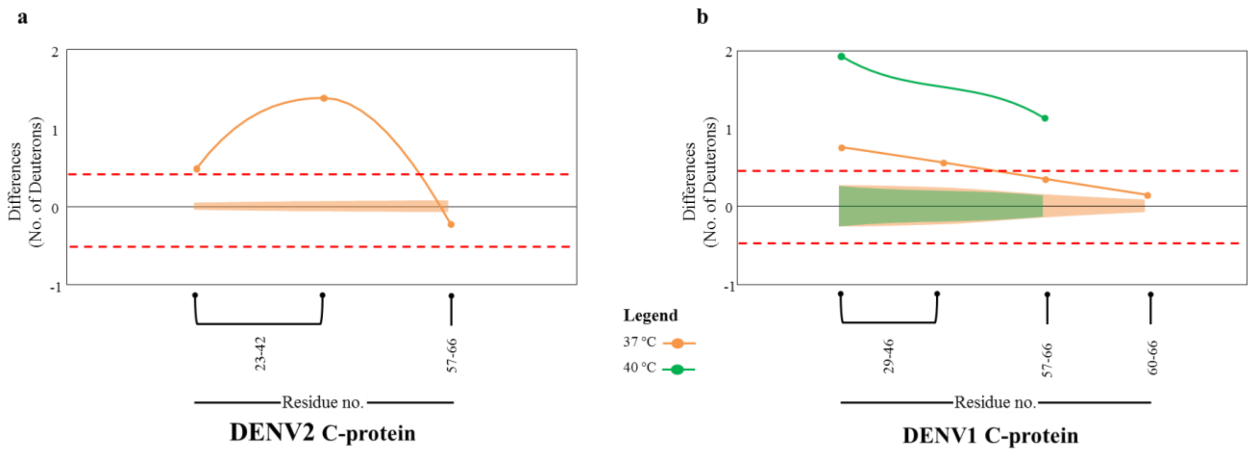

Supplementary Figure 7: Temperature-dependent increases in deuterium uptake observed in C-protein from both DENV serotypes. Deuterium exchange difference plots of peptides from C-protein from **a)** DENV2 and **b)** DENV1 listed from N- to C-terminus after 1 min of deuterium exchange at 37 and 40 °C relative to that at 28 °C (37/40 °C minus 28 °C). No peptides corresponding to DENV2 C-protein after 1 min of deuterium exchange at 40 °C were detected. Differences in deuterium exchange above 0.5 Da are considered significant (red dashed line). Standard error for each peptide is shown as overlapping shaded regions along the X-axis according to the difference plot. The individual standard error of each conditions is calculated as standard deviations observed across all the HDXMS measurements from three independent HDXMS experiments. The standard error for a given peptide in the difference plot represents the sum of such single sigma standard deviations of each of the two conditions being compared.

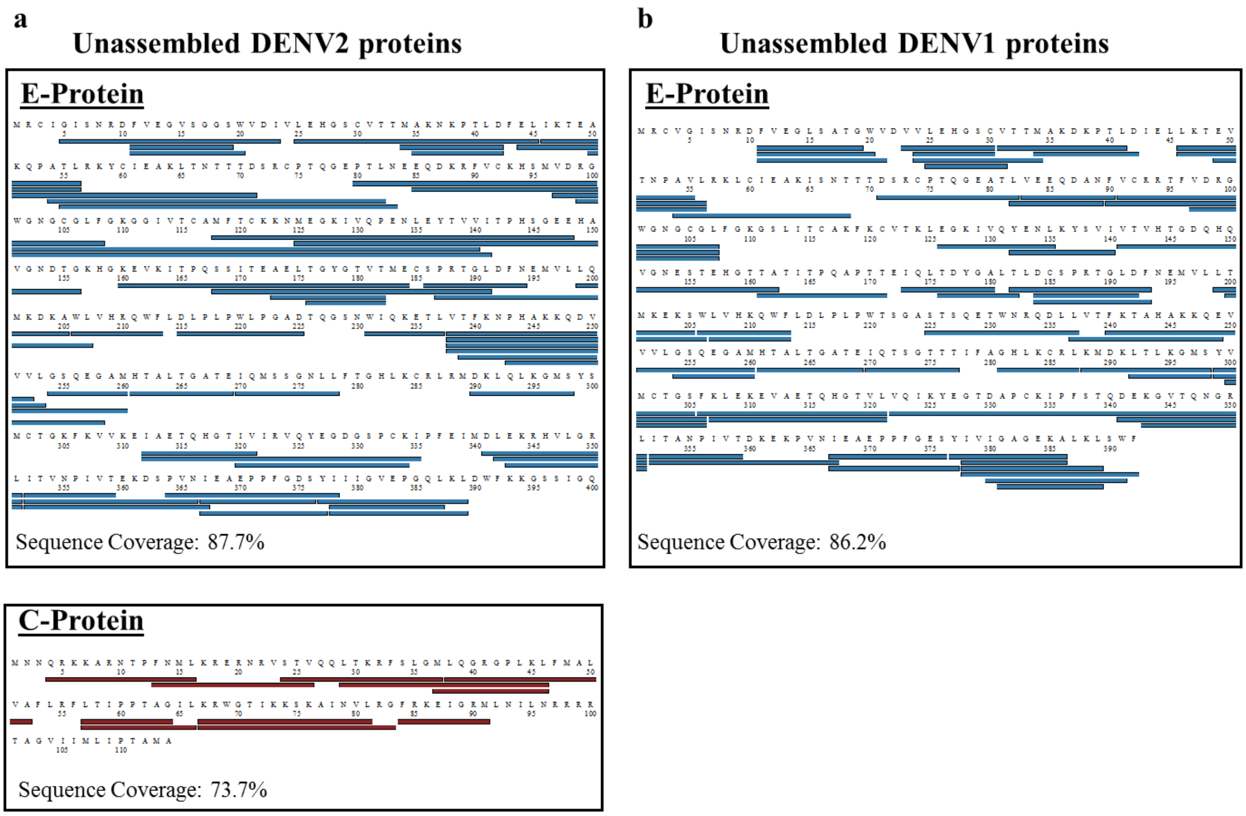

Supplementary Figure 8: **Primary sequence coverage map of pepsin fragmentation peptides from unassembled DENV2: C- and E-proteins and DENV1: E-protein.** Pepsin proteolyzed peptides of unassembled C- and E-proteins from DENV2 **(a)** and E-protein from DENV1 **(b)** are represented by blue and red bars for E- and C-proteins, respectively. Unassembled C- and E-proteins could only be expressed stably as deletion mutants: C-protein (1-100) from DENV2 and unassembled E-protein (1-394) from DENV2 and DENV1.

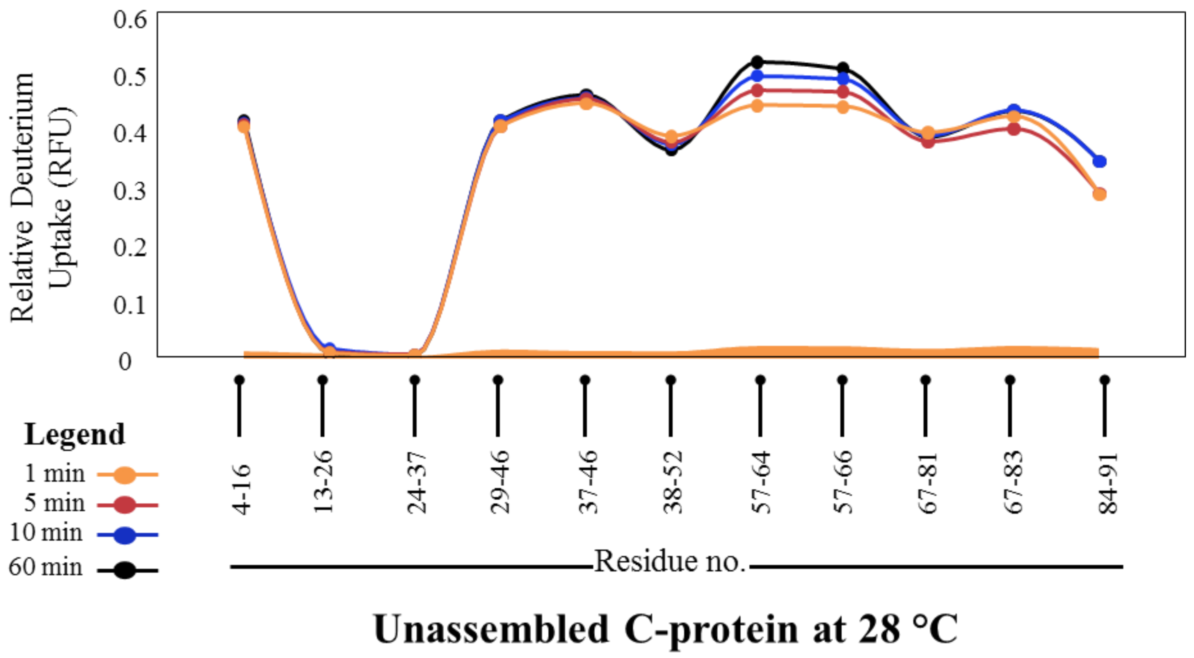

Supplementary Figure 9: Time-dependent changes in deuterium exchange observed in pepsin proteolyzed peptides from unassembled C-protein at 28 °C. Pepsin proteolyzed peptides from unassembled C-proteins are represented from N- to C-terminus. Deuterium uptake of each peptide for deuterium labelling time-points 1, 5, 10, and 60 min are represented as relative fractional deuterium uptake (RFU) values. Standard errors for each peptide are shown as shaded regions along the X-axis and are color coded same as RFU plots. The standard error for a given peptide represents the sum of the single sigma standard deviations over all the time-points from three independent HDXMS measurements.

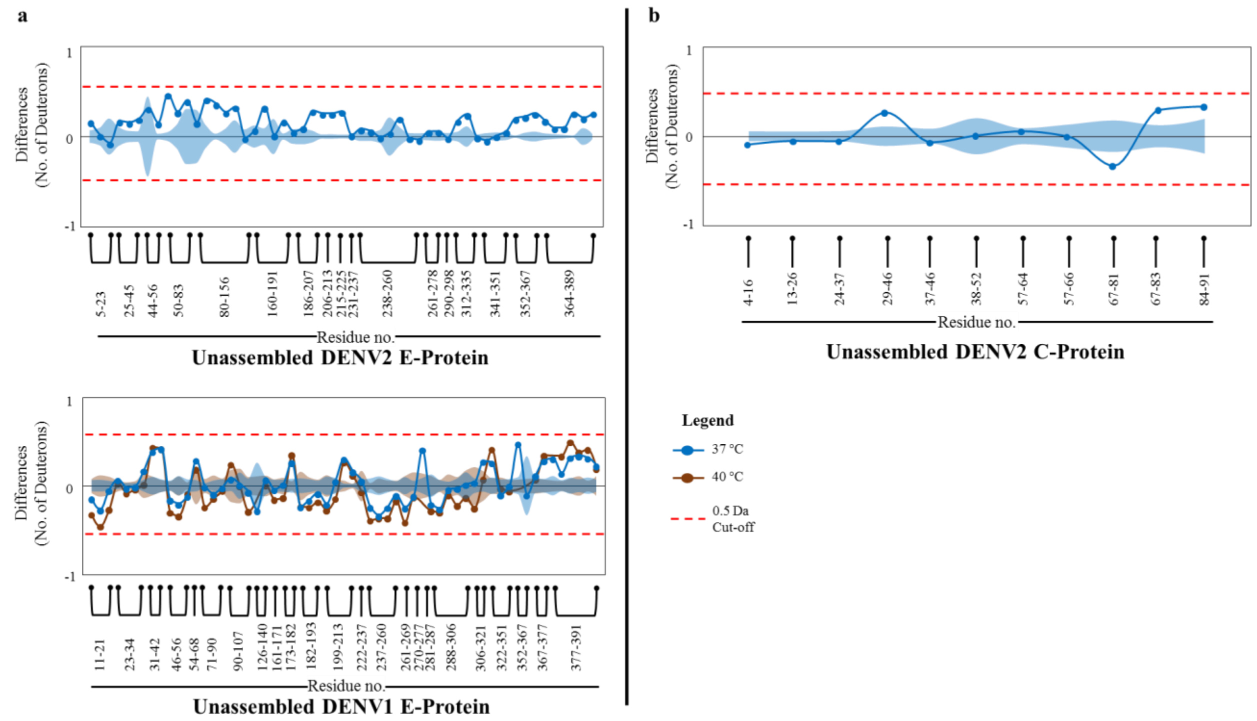

Supplementary Figure 10: Deuterium exchange in unassembled E and C-proteins are temperature-independent. a) Differences in number of deuterons after 1 min of deuterium exchange across pepsin fragmentation peptides for DENV2 unassembled E-protein between 37 and 28 °C and DENV1 unassembled E-protein between 37/ 40 and 28 °C after 1 min of deuterium exchange are represented in difference plots. **b)** Difference in number of deuterons exchange across pepsin fragmentation peptides for unassembled C-protein between 37 and 28 °C after 1 min of deuterium exchange are represented in a difference plot. A difference in deuterium exchange of 0.5 D is considered significant and represented by a red dashed line. None of the peptides from both unassembled E- and C-proteins showed any temperature-dependent differences in deuterium exchange greater than 0.5 D. Standard error for each peptide is shown as overlapping shaded regions along the X-axis colored according to the difference plots. The individual standard error of each conditions is calculated as standard deviations observed across all the HDXMS measurements from three independent HDXMS experiments. The standard error

for a given peptide in the difference plot represents the sum of such single sigma standard deviations of each of the two conditions being compared.

a

b

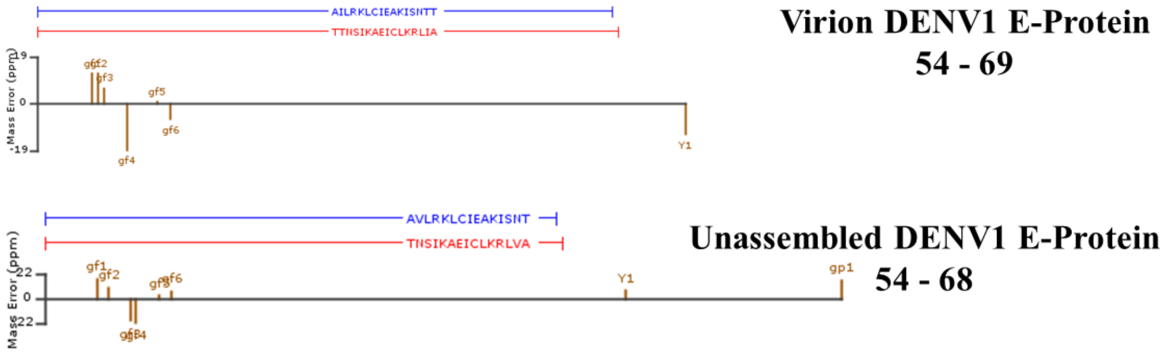

c

DENV2 (NGC) E-protein		Unassembled DENV2 E-protein	
**Residues**	**Glycans**	**Residues**	**Glycans**
57 - 69	gf1gf2gf4gf3gf5Y1gp1gp2	55 - 70	gf1gf2gf3gf4Y1gp1gp2
122 - 154	y3°Y1gf1gf2gf3gf4gf5gf6		
152 - 163	y6Y1gp1gp2gp3gf1gf2gf3gf5		

DENV1 (PVP 159) E-protein		Unassembled DENV1 E-protein	
**Residues**	**Glycans**	**Residues**	**Glycans**
54 - 69	gf1gf2gf3gf5gf4gf6Y1	54 - 68	gf1gf2gf5gf3gf4gf6Y1gp1
64 - 82	gf1gf2gf3gf5gf4gf6Y1gp1gp2gp3gp4		
152 - 173	b14°Y1gp1gp2gp3gp4gf1gf2gf3gf4gf5gf6		

MS^E Glycopeptide identification

Supplementary Figure 11: Fragmentation of glycopeptide in virion E-protein and unassembled E protein by MS^E. Fragmentation of glycan from a representative E-protein glycopeptide from intact DENV1 **(a),** unassembled E-protein from DENV1 **(b)** and Glycan fragmentation profile for all glycopeptides from intact DENV1 and DENV2 and unassembled DENV1 and DENV2 E-protein **(c)**.

Supplementary Tables:

Residues	DENV2 E-protein at 28 °C 1 min		DENV2 E-protein at 37 °C 26 s		DENV2 E-protein at 37 °C 1 min		Temperature correction for intrinsic deuterium exchange
	Uptake	Standard Deviation	Uptake	Standard Deviation	Uptake	Standard Deviation	$D_{37,26\,s} - D_{28,1\,min}$
^{4}IGISNRD^{10}	1.7	0.0	1.9	0.1	1.9	0.0	0.2
^{11}FVEGVSGGS^{19}	3.4	0.1	3.2	0.1	3.2	0.4	-0.2
^{11}FVEGVSGGSW^{20}	3.5	0.0	3.7	0.1	3.6	0.1	0.2
^{21}VDIVLEHGSC^{30}	0.7	0.0	1.3	0.1	1.3	0.1	0.6
^{31}VTTMAKNKPTL^{41}	1.7	0.0	1.4	0.1	1.7	0.0	-0.3
^{31}VTTMAKNKPTLD^{42}	1.8	0.1	1.9	0.1	1.9	0.1	0.1
^{31}VTTMAKNKPTLDF^{43}	1.7	0.0	1.9	0.1	1.8	0.0	0.2
^{32}TTMAKNKPTLD^{42}	1.5	0.1	1.6	0.1	1.7	0.0	0.1
^{33}TMAKNKPTLD^{42}	1.2	0.0	1.3	0.1	1.4	0.0	0.1
^{57}RKYCIEAKLTNTT^{69}	6.8	0.2	6.8	0.2	6.7	0.0	0.0
^{57}RKYCIEAKLTNTT^{69}	6.6	0.1	6.9	0.0	6.7	0.0	0.3
^{78}GEPSLNEEQDKRFVCKHSMVDRGW^{101}	3.9	0.1	4.6	0.2	4.5	0.0	0.7
^{152}GNDTGKHGKEIK^{163}	5.6	0.1	5.7	0.1	5.6	0.0	0.1
^{176}TGYGTVT^{182}	0.7	0.0	1.1	0.1	1.2	0.0	0.4
^{176}TGYGTVTM^{183}	1.1	0.0	1.4	0.0	1.5	0.0	0.3
^{184}ECSPRTGLD^{192}	2.5	0.1	2.2	0.1	2.3	0.1	-0.3
^{199}LQMENKA^{205}	1.7	0.1	2.1	0.3	2.1	0.0	0.4
^{202}ENKAWLVHRQWF^{213}	1.1	0.1	1.5	0.1	1.5	0.1	0.4
^{206}WLVHRQWF^{213}	0.8	0.0	1.0	0.1	1.0	0.1	0.2
^{207}LVHRQWF^{213}	0.7	0.1	1.0	0.1	0.8	0.0	0.3
^{238}VTFKNPHAKKQDVVV^{252}	3.0	0.0	3.8	0.1	3.9	0.1	0.8
^{238}VTFKNPHAKKQDVVVLGSQEGAM^{260}	4.7	0.2	6.8	0.2	6.6	0.0	2.1
^{261}HTALTGATE^{269}	1.6	0.0	2.6	0.2	2.8	0.1	1.0
^{270}IQMSSGNLL^{278}	2.5	0.1	3.1	0.1	2.8	0.0	0.6
^{280}TGHLKCRL^{287}	1.1	0.0	1.2	0.1	1.1	0.1	0.1
^{299}YSMCTGKF^{306}	1.5	0.0	1.3	0.0	1.4	0.0	-0.2
^{307}KVVKEIAETQHGTIV^{321}	1.7	0.1	2.3	0.2	2.2	0.0	0.6
^{322}IRVQYEGDGSPCKIPFE^{338}	1.7	0.1	2.5	0.0	2.4	0.1	0.8
^{352}ITVNPIVT^{359}	1. 3	0.1	1.2	0.0	1.1	0.0	-0.1
^{352}ITVNPIVTEKDSPVN^{366}	2.7	0.1	2.5	0.1	2.5	0.0	-0.2
^{352}ITVNPIVTEKDSPVNI^{367}	2.7	0.2	2.5	0.2	2.5	0.2	-0.2
^{352}ITVNPIVTEKDSPVNIEAEPPFGDS^{376}	3.3	0.2	2.9	0.0	3.0	0.1	-0.4
^{352}ITVNPIVTEKDSPVNIEAEPPFGDSY^{377}	3.1	0.1	2.9	0.1	2.7	0.1	-0.2
^{360}EKDSPVNIEAEPPFGDSY^{377}	1.6	0.1	1.6	0.1	1.6	0.0	0.0
^{367}IEAEPPFGDSY^{377}	1.1	0.1	1.2	0.2	1.1	0.4	0.1
^{378}IIIGVEPGQL^{387}	1.6	0.1	1.6	0.1	1.5	0.0	0.0
^{378}IIIGVEPGQLKL^{389}	2.1	0.1	2.1	0.1	1.9	0.1	0.0
^{378}IIIGVEPGQLKLNW^{391}	2.4	0.1	2.2	0.1	2.2	0.1	-0.2
^{392}FKKGSSIGQM^{401}	2.3	0.1	2.4	0.1	2.4	0.0	0.1
^{431}SIGKALHQVFGAIYGAAF^{448}	2.7	0.0	3.2	0.1	3.3	0.0	0.5

Supplementary Table 1: Comparison of deuterium exchange measurements on DENV2 labelled for 26 s and 1 min at 37 ºC with that of 1 min labelling at 28 ºC. Peptides showing temperature-dependent changes with DENV2 expansion (highlighted in orange). Examination of the differences in deuterium exchange in all peptides not showing temperature-dependent deuterium exchange between DENV2 labelled for 26 s at 37 °C with that for 1 min at 28 °C,

revealed differences of ±0.4 deuterons and established that the estimated 2.3-fold effect of temperature are within our standard error of our deuterium exchange measurements and experimental conditions.

Residue	DENV2 deuterium uptake			Residue	DENV1 deuterium uptake		
	28 °C	37 °C	40 °C		28 °C	37 °C	40 °C
^{11}FVEGVSGGS^{19}	3.3	3.4	3.0	^{11}FVEGLSATG^{19}	2.0	2.0	2.9
^{11}FVEGVSGGSW^{20}	2.7	2.9	2.7	^{11}FVEGLSATGW^{20}	2.1	2.2	3.1
^{31}VTTMAKNKPTL^{41}	1.4	1.5	1.5	^{31}VTTMAKDKPTL^{41}	0.6	0.6	1.2
^{31}VTTMAKNKPTLD^{42}	1.4	1.6	1.6	^{31}VTTMAKDKPTLD^{42}	0.7	0.7	1.3
^{176}TGYGTVTM^{183}	1.2	1.6	1.8	^{176}TDYGALTL^{183}	0.5	0.5	
^{184}ECSPRTGLD^{192}	2.3	2.4		^{184}DCSPRTGLD^{192}	1.5	1.4	1.5
^{202}ENKAWLVHRQWF^{213}	0.8	1.1	1.3	^{202}KEKSWLVHKQWF^{213}	0.2	0.2	1.0
^{206}WLVHRQWF^{213}	0.5	0.7	0.8	^{206}WLVHKQWF^{213}	0.1	0.2	
^{214}LDLPLPWLPGADTQGSN^{230}	1.3	1.2	1.3	^{214}LDLPLPWTSGASTSQET^{230}	0.7	0.7	2.1
^{238}VTFKNPHAKKQDVVVLGSQEGAM^{260}	4.0	6.5	6.0	^{238}VTFKTAHAKKQEVVVLGSQEGAM^{260}	2.7	2.9	3.8
^{251}VVLGSQEGAM^{260}	1.3	2.7	2.4	^{251}VVLGSQEGAM^{260}	0.8	0.7	1.9
^{261}HTALTGATE^{269}	1.3	2.8	2.6	^{261}HTALTGATE^{269}	0.9	0.9	1.8
^{299}YSMCTGKF^{306}	1.4	1.4	1.1	^{299}YVMCTGSF^{306}	0.8	0.9	
^{307}KVVKEIAETQHGTIV^{321}	1.9	2.9	3.1	^{307}KLEKEVAETQHGTVL^{321}	1.1	1.2	2.7
^{352}ITVNPIVTEKDSPVN^{366}	2.3	2.5	2.3	^{352}ITANPIVTDKEKPVN^{366}	1.1	1.0	
^{352}ITVNPIVTEKDSPVNI^{367}	2.7	2.8	2.4	^{352}ITANPIVTDKEKPVNI^{367}	0.9	0.8	2.0
^{352}ITVNPIVTEKDSPVNIEAEPPFGDS^{376}	2.9	3.2	3.1	^{352}ITANPIVTDKEKPVNIEAEPPFGES^{376}	1.6	1.4	2.8
^{352}ITVNPIVTEKDSPVNIEAEPPFGDSY^{377}	2.8	3.0	3.1	^{352}ITANPIVTDKEKPVNIEAEPPFGESY^{377}	1.4	1.3	2.9
^{367}IEAEPPFGDSY^{377}	1.0	1.2	1.2	^{367}IEAEPPFGESY^{377}	0.7	0.8	1.5
^{392}FKKGSSIGQM^{401}	1.9	2.4	2.4	^{392}FKKGSSIGKM^{401}	0.6	0.7	1.5

Supplementary Table 2: Comparison of deuterium uptake in equivalent pepsin fragment peptides from DENV2 and DENV1 at 28, 37 and 40 °C. Peptides from DENV2 and DENV1 are colored red and blue respectively. Differences in sequence between DENV2 and DENV1 are colored black. Grey boxes represent peptides undetected at 40 °C.

DENV2 E-protein peptide		DENV2 E-protein at 28 °C			Unassembled DENV2 E-protein at 28 °C			Differences
	Undeuterated Spectral Width	Deuterium uptake*	SD	Spectral Width	Deuterium uptake*	SD	Spectral Width	
^{11}FVEGVSGGS^{19}	4	3.3	0.1	10	3.5	0.1	10	0.2
^{11}FVEGVSGGSW^{20}	4	2.7	0.0	10	4.2	0.0	10	1.5
^{176}TGYGTVT^{182}	3	1.2	0.0	6	2.4	0.0	7	1.2
^{199}LQMENKA^{205}	4	1.8	0.0	7	2.7	0.0	8	0.9
^{206}WLVHRQWF^{213}	4	0.5	0.0	6	1.2	0.0	6	0.7
^{238}VTFKNPHAKKQDVVV^{252}	6	2.8	0.1	9	4.9	0.1	12	2.1
^{238}VTFKNPHAKKQDVVVLGSQEGAM^{260}	7	4.0	0.2	15	8.7	0.1	19	4.7
^{261}HTALTGATE^{269}	4	1.3	0.1	7	3.9	0.0	9	2.6
^{270}IQMSSGNLL^{278}	5	1.5	0.1	8	3.8	0.0	8	2.3
^{352}ITVNPIVT^{359}	4	1.2	0.1	6	2.9	0.0	6	1.7
^{352}ITVNPIVTEKDSPVN^{366}	5	2.3	0.2	10	5.2	0.0	11	2.9
^{352}ITVNPIVTEKDSPVNI^{367}	4	2.7	0.1	9	5.1	0.0	11	2.4
^{367}IEAEPPFGDSY^{377}	5	1.0	0.1	7	2.0	0.0	8	1.0
^{378}IIIGVEPGQL^{387}	4	1.5	0.2	7	2.4	0.0	7	0.9
^{378}IIIGVEPGQLKL^{389}	5	2.2	0.3	9	3.3	0.0	9	1.1

Supplementary Table 3: Spectral width of analysis of peptides common to DENV2 E-protein and unassembled DENV2 E-protein at 28 °C.

DENV1 E-protein peptide		DENV1 E-protein at 28 °C			Unassembled DENV1 E-protein at 28 °C			Differences
	Undeuterated Spectra width	Deuterium uptake*	SD	Spectral Width	Deuterium uptake*	SD	Spectral Width	
^{11}FVEGLSATG^{19}	4	2.0	0.0	8	3.6	0.0	9	1.6
^{11}FVEGLSATGW^{20}	5	2.1	0.0	8	4.0	0.0	11	1.9
^{23}VVLEHGSC^{30}	4	0.2	0.0	5	0.8	0.0	6	0.6
^{24}VLEHGSC^{30}	4	0.2	0.0	5	0.7	0.1	5	0.5
^{25}LEHGSCV^{31}	4	0.1	0.1	4	0.6	0.1	7	0.5
^{31}VTTMAKDKPTL^{41}	5	0.6	0.0	6	2.6	0.2	9	2.0
^{90}FVCRRTFVDRGWGNGCGL^{107}	6	0.6	0.0	7	3.6	0.0	15	3.0
^{91}VCRRTFVDRGWGNGCGL^{107}	6	1.0	0.1	9	4.3	0.1	13	3.3
^{126}EGKIVQYENL^{135}	4	1.3	0.0	8	3.2	0.1	10	1.9
^{132}YENLKYSVI^{140}	4	0.2	0.0	5	0.9	0.1	6	0.7
^{184}DCSPRTGLD^{192}	4	1.5	0.1	9	2.2	0.1	9	0.7
^{206}WLVHKQWF^{213}	4	0.1	0.0	5	0.9	0.0	6	0.8
^{251}VVLGSQEGAM^{260}	4	0.8	0.1	7	3.4	0.1	10	2.6
^{261}HTALTGATE^{269}	4	0.9	0.0	7	3.1	0.0	9	2.2
^{299}YVMCTGSF^{306}	4	0.8	0.1	6	3.4	0.0	9	2.6
^{307}KLEKEVAETQHGTVL^{321}	5	1.1	0.0	8	4.4	0.1	13	3.3
^{352}ITANPIVTDKEKPVNI^{367}	5	0.9	0.2	6	3.2	0.1	11	2.3
^{367}IEAEPPFGESY^{377}	4	0.7	0.1	6	2.3	0.2	8	1.6

Supplementary table 4: Spectral width of analysis of DENV1 E-protein and unassembled DENV1 E-protein at 28 °C.

Supplementary Note 1

Optimization of pepsin cleavage efficiency and deuterium exchange times for HDXMS of intact DENV particles

We modified standard pepsin digestion and quench conditions for HDXMS globular protein analysis with additional steps for improved pepsin cleavage of the more stable viral particles compared to proteins in solution and to exclude lipid bilayer components from signal interference with peptides. Intact viral particles were isolated and purified to a concentration of 0.25 mg/ml as described in materials and methods. Deuterium exchange of DENV2 was initiated by 10-fold dilution in NTE buffer reconstituted in D_2O, resulting in a final concentration of 90% deuterium in the reaction. HDXMS time course analyses were initially carried out at 28 and 37 °C (in a scan across time points 0, 1, 5, 10, 60 min). Our optimized experimental conditions included 1.5 M guanidine HCl and 0.25 M TCEP-HCl in the reaction quench mixture at pH 2.5. Separation of lipids from the viral envelope prior to mass spectrometry analysis was achieved by TiO_2 incubation [1]. We subjected the DENV2 samples to offline digestion using immobilized pepsin beads for 5 min and the samples were injected into nano-UPLC HDX sample manager (Waters, Milford, MA) as described [2]. Peptides were ionized by electrospray into a SYNAPT G2-Si mass spectrometer (Waters, Milford, MA) acquiring in MS^E mode for detection and mass-measurements.

Sequencing of the C-, E- and M-pepsin fragment peptides from this initial offline pepsin digested samples yielded a sequence coverage of 66.7% for E-protein while C and M-pepsin fragment peptides were undetected. The relative deuterium exchange across all the pepsin fragment peptides for E-protein from DENV2 is represented in a difference plot in Supplementary Fig. 3. We observed that most peptides showed only small magnitude time-

dependent increases in exchange across the time series from t=1 min to 60 min (Supplementary Fig. 3). This suggested that 1 min of deuterium exchange represented an optimal time-point where most of the exchange had already occurred, for monitoring deuterium exchange and probe temperature perturbations on whole DENV2 viral particles.

In order to detect C- and M-proteins and also improve the sequence coverage of E-protein, we further optimized pepsin cleavage conditions by using an online pepsin column (Enzymate, Waters) that offered improved digestion under high pressure. Sequencing of C, E and M-proteins from online pepsin digestion, subsequently yielded higher sequence coverages of 20%, 77.2% and 56% respectively (Supplementary Fig. 2a i, ii, iii). All results mapping deuterium exchange changes on the structure of E-protein are derived from samples subjected to deuterium exchange time of 1 min from online pepsin proteolysis (Figs. 2, 3, 5). This indicated that modifications in digestion protocols from digestion with offline pepsin beads to online high pressure column significantly improved sequence coverage of E-protein and also enhanced the detection and identification of peptides from the smaller sized C and M-proteins. Therefore, all samples were subsequently digested with online pepsin column.

Supplementary Note 2

Temperature effects on HDX rates on model peptides

We then set out to examine effects of increased temperature (37 °C) to monitor temperature-dependent expansion in DENV2 [3]. The effects of temperature, salt concentrations and amino acid composition on the intrinsic rate of exchange in model peptides have been extensively demonstrated [4]. This together with an online calculator developed by the research group of S. W. Englander (http://hx2.med.upenn.edu/download.html), MS-excel based calculator

for Deuterium exchange in H_2O) [4, 5], allowed us to estimate the increase in rates of intrinsic exchange when the temperature was shifted by 9 °C, from 28 to 37 °C. The calculator predicts a 2.3X increase in intrinsic rates of exchange at all backbone amide positions in a model Ala dipeptide at 37 °C. We predicted that for our intact protein and viral particle peptides, under our HDXMS experimental conditions, this 2.3X magnitude increase in intrinsic rates of deuterium exchange would be insignificant as it is a much smaller change compared to the intrinsic rates of exchange. At deuterium exchange times of 1 min and longer, these differences would be practically undetectable. To experimentally confirm this, we carried out test studies with HDXMS on post-pepsin hydrolyzed unassembled DENV2 E-protein. We were unable to detect observable differences in centroid values between 28 and 37 °C across all peptides (Supplementary Fig. 5 a, b). This is attributable to slower conformational changes rather than faster intrinsic rates of deuterium exchange which govern the experimentally observed deuterium exchange rates. The changes in deuterium exchange due to conformational changes were significantly greater than the 2.3X increases in deuterium exchange at our experimental time points of 1 min and longer.

In addition to assessing the effects of increased temperature on pre-pepsinized peptides of E-protein from DENV2, we also accounted for the effects of temperature on whole DENV2 particles by conducting the deuterium exchange of viral particles at 37 °C for 26 s. The deuterium exchange time of 26 s was rationalized from division of 2.3 from 60 s (1 min) to account for the calculated 2.3 fold increase in intrinsic deuterium exchange at 37 °C as compared to 28 °C. Deuterium uptake for all peptides after 26 s or 1 min of deuterium exchange at 37 °C were largely similar, with differences less than 0.4 deuterons (Supplementary Table 1, Supplementary Fig. 5). Differences in deuterium uptake between 26 s or 1 min at 37 °C and 1

min at 28 °C for all E-protein pepsin proteolyzed peptides were calculated and represented on a difference plot (Supplementary Fig. 5). Peptides spanning loci that exhibit temperature-dependent increase in deuterium uptake were identical between DENV2 at 37 °C after deuterium exchange of 26 s or 1 min. Furthermore, the observed deuterium uptake variation of 0.4 D between the deuterium exchange time of 26 s and 1 min of DENV2 at 37 and 28 °C respectively, which we reasoned to be due to the 2.3 fold increase in intrinsic deuterium exchange rate, was within the 0.5 D significance threshold fordeuterium uptake differences. Our experimental conditions would therefore report predominantly on temperature-induced conformational changes across the capsid proteome of intact DENV2/1 particles together with unassembled C- and E-proteins.

Supplementary References

1. Hebling CM, Morgan CR, Stafford DW, Jorgenson JW, Rand KD, Engen JR. Conformational analysis of membrane proteins in phospholipid bilayer nanodiscs by hydrogen exchange mass spectrometry. *Anal Chem* **82**, 5415-5419 (2010).

2. Wales TE, Fadgen KE, Gerhardt GC, Engen JR. High-speed and high-resolution UPLC separation at zero degrees Celsius. *Anal Chem* **80**, 6815-6820 (2008).

3. Fibriansah G, *et al.* Structural Changes in Dengue Virus When Exposed to a Temperature of 37 degrees C. *J Virol* **87**, 7585-7592 (2013).

4. Bai Y, Milne JS, Mayne L, Englander SW. Primary structure effects on peptide group hydrogen exchange. *Proteins* **17**, 75-86 (1993).

5. Connelly GP, Bai Y, Jeng MF, Englander SW. Isotope effects in peptide group hydrogen exchange. *Proteins* **17**, 87-92 (1993).
